# Automated Manufacture of Autologous CD19 CAR-T Cells for Treatment of Non-hodgkin Lymphoma

**DOI:** 10.3389/fimmu.2020.01941

**Published:** 2020-08-07

**Authors:** Zachary Jackson, Anne Roe, Ashish Arunkumar Sharma, Filipa Blasco Tavares Pereira Lopes, Aarthi Talla, Sarah Kleinsorge-Block, Kayla Zamborsky, Jennifer Schiavone, Shivaprasad Manjappa, Robert Schauner, Grace Lee, Ruifu Liu, Paolo F. Caimi, Ying Xiong, Winfried Krueger, Andrew Worden, Mike Kadan, Dina Schneider, Rimas Orentas, Boro Dropulic, Rafick-Pierre Sekaly, Marcos de Lima, David N. Wald, Jane S. Reese

**Affiliations:** ^1^Department of Pathology, Case Western Reserve University, Cleveland, OH, United States; ^2^Department of Nutrition, Case Western Reserve University, Cleveland, OH, United States; ^3^The Alan Turing Institute, British Library, London, United Kingdom; ^4^Stem Cell Transplantation Program, University Hospitals Seidman Cancer Center, Cleveland, OH, United States; ^5^Department of Medicine, University Hospitals Cleveland Medical Center, Cleveland, OH, United States; ^6^Lentigen Technology, Inc., a Miltenyi Biotec Company, Gaithersburg, MD, United States; ^7^Department of Pediatrics, Seattle Children’s Research Institute, Seattle, WA, United States; ^8^Department of Pediatrics, University of Washington School of Medicine, Seattle, WA, United States; ^9^Department of Pathology, University Hospitals Cleveland Medical Center, Cleveland, OH, United States; ^10^Case Comprehensive Cancer Center, Case Western Reserve University, Cleveland, OH, United States

**Keywords:** automated, CAR-T, manufacturing, Prodigy, stem cell memory T

## Abstract

Chimeric antigen receptor T cells (CAR-T cell) targeting CD19 are effective against several subtypes of CD19-expressing hematologic malignancies. Centralized manufacturing has allowed rapid expansion of this cellular therapy, but it may be associated with treatment delays due to the required logistics. We hypothesized that point of care manufacturing of CAR-T cells on the automated CliniMACS Prodigy^®^ device allows reproducible and fast delivery of cells for the treatment of patients with non-Hodgkin lymphoma. Here we describe cell manufacturing results and characterize the phenotype and effector function of CAR-T cells used in a phase I/II study. We utilized a lentiviral vector delivering a second-generation CD19 CAR construct with 4-1BB costimulatory domain and TNFRSF19 transmembrane domain. Our data highlight the successful generation of CAR-T cells at numbers sufficient for all patients treated, a shortened duration of production from 12 to 8 days followed by fresh infusion into patients, and the detection of CAR-T cells in patient circulation up to 1-year post-infusion.

## Introduction

Genetically engineered T lymphocytes expressing a chimeric antigen receptor (CAR-T cells) are immune cells designed to lyse tumor cells upon CAR ligation with tumor-specific antigens. CD19 CAR-T cells are engineered to recognize CD19-expressing B cell malignancies and two CAR-T products are FDA approved for the treatment of relapsed/refractory patients with non-Hodgkin lymphoma (NHL) (1). Approximately 50–70% of NHL patients receiving CD19 CAR-T cells achieve complete remission (2). Numerous preclinical studies and clinical trials are currently evaluating this therapy in additional contexts including solid tumors (3).

Current practice in the manufacture of CAR-T cells requires a series of hands-on steps that can often take 2 weeks to achieve a clinical cell dose. Upon collection of peripheral blood leukapheresis products from patients, T cells are isolated, expanded using anti-CD3/CD28 co-stimulation and cytokine supplementation, transduced with a CAR-encoding vector, and harvested for cryopreservation prior to infusion. Numerous quality control mechanisms are built in to ensure sample purity and tumor-induced effector activity. In attempts to optimize patient outcomes, several protocols have been designed to generate a mix of phenotypes in the final CAR-T product. In general, naive, memory, and stem-like phenotypes have shown the highest potential for achieving a long-lasting response, and CAR-T manufacture processes are designed to enrich for such phenotypes (4–8). Conditions that vary between protocols include the specific cytokines used, dose of cytokines, duration of expansion, and freeze-thaw conditions. The ultimate goal of CAR-T cell manufacturing is to deliver a highly cytotoxic product that exhibits high proliferative capacity, maintains long-term memory, is resistant to exhaustion, and does not produce cytokine release syndrome (CRS).

As the field rapidly progresses, there is a need for a standardized, sterile, reproducible and scalable protocol for clinical-grade manufacturing of CAR-T cells (9). While several methods are available, one platform, the CliniMACS Prodigy^®^, offers a closed automated system capable of manufacturing CAR-T cells outside of a cleanroom environment in an unclassified space while maintaining Good Manufacturing Practice (GMP) compliance (10). This technology not only increases the accessibility of CAR-T cell therapy to centers lacking sophisticated cell processing capabilities, but it is also adaptable to the needs of individual samples, offers sample monitoring, enables delivery of non-frozen cells, and rapidly yields a functional product within 2 weeks (11–13). Here, we demonstrate sufficient *ex vivo* expansion of CAR-T cells in a timespan as short as 8 days, a significant finding in consideration of a previous report that indicates there is improved anti-leukemic activity of CAR-T cells with shorter culture durations (14).

Also, we report the successful generation and infusion of autologous CD19 CAR-T cells in heavily pre-treated patients (15), and document enrichment of memory or stem-like qualities, which have been associated with improved patient response in other studies (16, 17). Specifically, we observed higher frequencies of T cell central memory (TCM) and stem cell memory (TSCM) phenotypes and cells expressing transcription factors TBET and GATA3. The CAR-T cells effectively killed CD19^+^ cells *in vitro* and *in vivo* while maintaining polyfunctionality. We in addition observed a potential correlation between quality control potency assays and patient response rates. Moreover, by use of a commercially available, fluorescently labeled CD19 peptide we employed a method to measure circulating CD19 CAR T cells from patient peripheral blood by flow cytometry that correlates well with peripheral blood vector copy number.

## Materials and Methods

### Cohort Description

Clinical leukapheresis products were obtained from NHL patients undergoing CAR-T cell therapy at University Hospitals Seidman Cancer Center under a phase I/II study (ClinicalTrials.gov Identifier: NCT03434769; IND 17932). Mononuclear peripheral blood cell apheresis products were processed within 24 h of receipt without cryopreservation. The study was approved by the institutional review board and all patients gave written informed consent.

### CliniMACS Prodigy^®^ Settings

CAR-T cells were manufactured within the CliniMACS Prodigy^®^ (Miltenyi Biotec, Bergisch Gladbach, Germany) device using the TCT software program and TS520 tubing set (Miltenyi Biotec, Bergisch Gladbach, Germany). The main components of the Prodigy^®^ and the instrument setup followed the protocol given by Miltenyi Biotec and were outlined in (13). All processing was performed at the Cellular Therapy Lab of University Hospitals Cleveland Medical Center Seidman Cancer Center/Case Western Reserve University Center for Regenerative Medicine.

### Lentiviral Vector

All experiments described in this paper used a novel CD19 CAR vector, LTG1563, which was developed and provided by Lentigen, a Miltenyi Biotec company (Gaithersburg, MD, United States). The vector contains a scFv FMC63-based targeting domain, CD8-derived hinge region, TNFRSF19-derived transmembrane region, 4-1BB/CD137 costimulatory domain, and CD3-zeta chain intracellular signaling domain.

### Cell Culture Reagents

Clinical-grade reagents used to manufacture the CAR-T cells included: CliniMACS Buffer, TexMACS Media, CliniMACS CD4 reagent, CliniMACS CD8 reagent, TransAct, and the cytokines IL-7 and IL-15 (Miltenyi Biotec, Bergisch Gladbach, Germany). Reagents were utilized according to manufacturer’s instructions. One 25 μg vial each of IL-7 and IL-15 was added per 2L bag of media. A 25% stock solution of Human Serum Albumin (HSA) was used to supplement the CliniMACS Buffer to a concentration of 0.5%. Human AB serum was used to supplement the TexMACS Media to a concentration of 3% and was from Innovative Research (Novi, MI, United States). TexMACS media was supplemented with Human AB serum for 6 days of the cell culture and then replaced with media without Human AB serum for the duration of the culture.

### Flow Cytometry

Prior to and post-CD4/CD8 enrichment, T cells were phenotyped with CD4 VioGreen, CD8 APC-Vio770, CD45 VioBlue, and 7AAD (reagents from Miltenyi Biotec, Bergisch Gladbach, Germany). Final CAR-T products after harvest from the Miltenyi Prodigy^®^ were phenotyped with CD19 Fc Chimera protein (R&D Systems, Minneapolis, MN, United States) followed by the anti-Fc-PE secondary antibody (Jackson ImmunoResearch, West Grove, PA, United States) as described previously (18), CD45 VioBlue, CD4 VioGreen, CD8 APC-Vio770, CD3 FITC, CD14 APC, CD20 PE-Vio770, CD56 PE, CD16 PE, and 7AAD (reagents from Miltenyi Biotec, Bergisch Gladbach, Germany) to establish the following populations: CD45^+^ lymphocytes, CD4^+^ T cells, CD8^+^ T cells, and CD4^+^CD8^+^ T cells, CD3^+^ T cells, CD14^+^ monocytes, CD20^+^ B cells, CD56^+^CD16^+^ NK cells, and CD3^+^CD56^+^CD16^+^ NK T cells.

Flow analysis for circulating CAR-T cells was performed using a FITC-labeled CD19 peptide (amino acids 20–291) (ACRObiosystems, Newark, DE, United States), CD3 PE, CD8 APC-Vio770, CD45 VioBlue, and 7AAD (Miltenyi Biotec, Bergisch Gladbach, Germany). All samples from the panels above were analyzed on the CliniMACS MACSQuant Flow Cytometer (Miltenyi Biotec, Bergisch Gladbach, Germany). BD’s 18-color Fortessa flow cytometer (BD Biosciences, Franklin Lakes, NJ, United States) was used to assess CAR-T cell phenotype. The following antibodies were used for surface staining: Recombinant Human CD19 Fc Chimera Protein (R&D Systems, Minneapolis, MN, United States), Anti Fc-PE (Jackson ImmunoResearch, West Grove, PA, United States), CD3 AF700 (BD Biosciences, Franklin Lakes, NJ, United States), CD4 BV605 (Biolegend, San Diego, CA, United States), CD8 BUV737 (BD Biosciences, Franklin Lakes, NJ, United States), CD19 BV510 (BD Biosciences, Franklin Lakes, NJ, United States), CD45RO BUV395 (BD Biosciences, Franklin Lakes, NJ, United States), CD45RA BV650 (Biolegend, San Diego, CA, United States), CD45 BV786 (BD Biosciences, Franklin Lakes, NJ, United States), CD95 FITC (BD Biosciences, Franklin Lakes, NJ, United States), CD27 BV711 (BD Biosciences, Franklin Lakes, NJ, United States), CCR7 PE Cy7 (BD Biosciences, Franklin Lakes, NJ, United States); and Thermo Fisher’s (Waltham, MA, United States) FOXP3 transcription factor buffer kit was used to intracellularly stain as per manufacturer’s protocol for TCF7 AF647 (Biolegend, San Diego, CA, United States), Tbet PerCPCy5.5 (BD Biosciences, Franklin Lakes, NJ, United States), and GATA3 PECF594 (BD Biosciences, Franklin Lakes, NJ, United States). Data was analyzed by FlowJo 10.4.2 software (Ashland, OR, United States). In addition to manual gating for conventional subsets, high-dimensional clustering techniques were used to identify unique cell clusters within CAR-T products. Here, the Phenograph clustering algorithm (19) (Rphenograph package in R; default settings where k was set to 20) was used on pre-gated live CD3 + T cells. The phenograph clusters were projected on a 2-dimensional UMAP (20) (umap-learn in R; default settings) and statistical assessment of alteration in cluster frequencies within CAR-T cells was done using GraphPad Prism (GraphPad Software, San Diego, CA, United States).

### Cytotoxicity Assay

Cytotoxicity assays were performed by co-culturing CAR-T cells with target cells at various effector to target ratios as previously reported (21). Briefly, target cells were pre-labeled with 1 μg/mL calcein-AM (Life Technologies, Carlsbad, CA, United States) for 30 min at 37°C. 10,000 target cells were plated per well of a 96-well plate and various ratios of CAR-T cells were added. After 4 h the cells were analyzed by flow cytometry to detect the loss of fluorescence in the target cells using the Attune NxT cytometer. Target cells used include Raji, Daudi, JVM2, and OCI-AML3 (all cell lines from ATCC, Manassas, VA, United States).

### Animal Study

Six to eight week old NOD.*Cg-Prkdc^*scid*^Il2rgt^*m*1*Wjl*^/SzJ* mice (NSG, The Jackson Laboratory, Bar Harbor, NE, United States) were injected *i.v.* with 1.0 × 10^6^ Raji-luciferase cells as previously described (18), with several modifications. Briefly, Raji-luciferase cells were injected into mice via tail vein. Mice were randomly split into 4 groups of 5 and received an *i.v.* injection of 2.5 × 10^6^ CAR-T cells (normalized to the transduction efficiency; non-transduced T cell control group received 8 × 10^6^ cells) 1 week after tumor cell injection. Disease progression was followed weekly by bioluminescence imaging using the IVIS Spectrum Imager (PerkinElmer, Waltham, MA, United States). Blood was collected at 2- and 4- weeks post CAR-T injection and analyzed by flow cytometry to track the circulating levels CAR-T cells. Mice were sacrificed at 5 or 6 weeks post CAR-T infusion. Blood, liver, kidney, spleen, bone marrow, and lymph nodes were isolated and cells were separated by homogenization with RBC lysis as needed, and then analyzed by flow cytometry.

### Cytokine Assay

To evaluate the levels of plasma cytokines in tumor-bearing NSG mice receiving CAR-T therapy, we used the U-PLEX assay (Meso Scale MULTI-ARRAY Technology) commercially available by MSD (Meso Scale Discovery Rockville, MD, United States). Using this platform, a cytokine panel was screened: TGFβ1, TGFβ2, IFN-α2a, IFNβ, IFNγ, TNFα, TRAIL, VEGF, IL-6, IL-9, IL-15, IL-21, IL-22, and IL-29. 25 μL of plasma from each donor was used following the manufacturer’s instructions. Electrochemiluminescence was detected using MESO QuickPlex SQ 120 (Meso Scale Discovery Rockville, MD, United States). The results were extrapolated from the standard curve from each specific analyte and plotted in pg/mL using the DISCOVERY WORKBENCH v4.0 software (Meso Scale Discovery, Rockville, MD, United States).

### Proviral Detection

DNA was isolated from whole mononuclear cells and probed for rev-responsive element with a Taq-based qPCR assay (Lentigen Corporation, Gaithersburg, MD, United States). DNA was measured with a CFX Multicolor Real-Time PCR Detection System (Bio-Rad Laboratories, Hercules, CA, United States). Copy number per CAR-T cell estimated by the formula (copy#/ng) × (0.006ng/cell)/transduction rate.

### Statistical Analysis

Unpaired two-tailed *t*-tests were used for comparison of two groups. One-way ANOVA was used when more than two groups were analyzed for statistical significance. All statistical analyses were done using R in R Studio 1.1.456^1^, GraphPad Prism 8.3.0 (GraphPad Software, San Diego, CA, United States) or Microsoft Excel 16.16.9^2^. Statistical significance was given as ^∗^, ^∗∗^, ^∗∗∗^, ^****^ by *p*-values less than <0.05, <0.01, <0.001, or <0.0001, respectively.

### Data Sharing Statement

For original data, please contact dnw@case.edu.

## Results

### Manufacturing Clinical Grade CD19 CAR-T Cells

**FIGURE 1:**
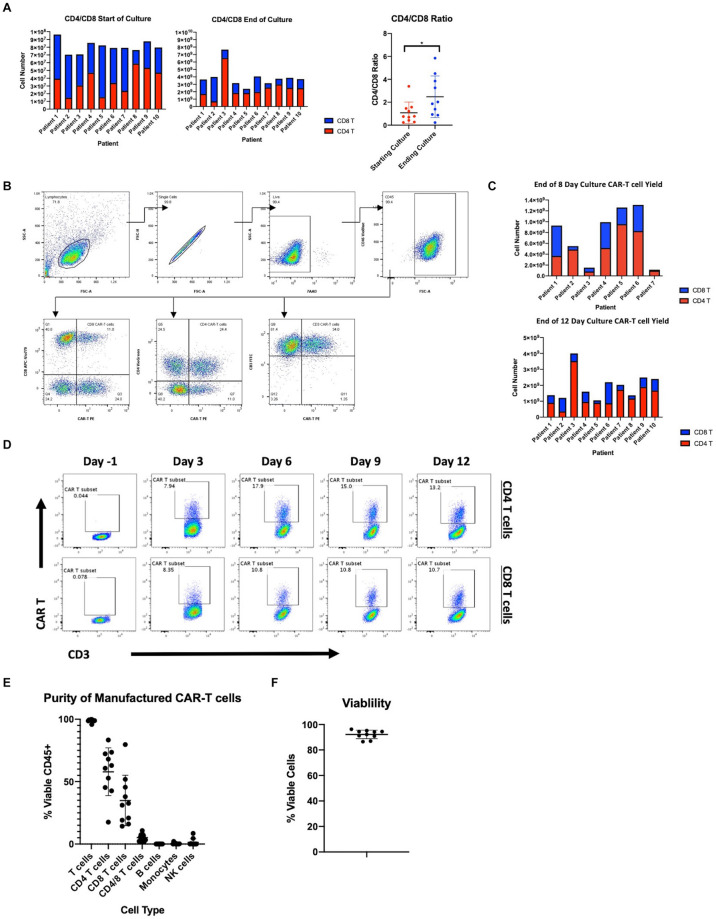
Manufacturing clinical grade CD19 CAR-T cells using the CliniMACS Prodigy. **(A)** Total cell numbers and CD4 and CD8 T cell ratios at the start of culture after CD4/CD8 enrichment and after 12 days of culture. **(B)** Flow cytometric gating strategy for identification of CD19 CAR expressing T cells. **(C)** Number of CAR-T cells after 8 or 12 days of culture and CD4 and CD8 T cell composition. **(D)** Time course of CAR transduction rates of CD3 T cells for duration of culture (*n* = 1). **(E)** Purity of final product after expansion in the Prodigy (*n* = 10). **(F)** Percent viable T cells of CD45^+^ cells after expansion in the Prodigy (*n* = 10). Statistical significance was given as *, **, ***, or **** by *p*-values less than < 0.05, < 0.01, < 0.001, or < 0.0001, respectively.

Concerns exist on the ability to consistently expand patient-derived T cells, as these cells are often functionally impaired due to prior chemotherapy as well as the primary disease process (15). Here we present data on the manufacturing of CAR19 LTG1563 from autologous T cells. The T cell composition of patient apheresis samples were 19.06% ± 10.06 CD4 T cells and 27.9% ± 14.15 CD8 T cells (Table 1). Each apheresis collection was loaded onto the CliniMACS Prodigy^®^ and column purified using the TS520 Tubing set and CliniMACS CD4 and CD8 reagents (microbeads). After column purification, CD4 and CD8 T cells were enriched to 37.85% ± 15.81 and 46.21% ± 15.88, respectively (Table 1). Interestingly, the expansion conditions led to a selective increase of CD4 T cells, increasing the average CD4:CD8 ratio from 1.03 post-CD4/CD8 enrichment to 2.48 by the end of culture (Figure 1A).

**TABLE 1:** Purity of apheresis and recovery post-enrichment of CD4 and CD8 cells.

	**% Viable CD45^+^ cells**	
**Parameter**	**Pre-column purity**	**Post-column recovery**
CD4	19.06 ± 10.06	37.85 ± 15.81
CD8	27.9 ± 14.15	46.21 ± 15.88

The CliniMACS Prodigy was loaded with ∼1 × 10^8^ CD3 cells and the cells expanded with anti-CD3/CD28 co-stimulation and IL-7/IL-15 supplementation. After culture, we achieved a median 41.1-fold expansion of CD3^+^ cells (range 30.0–78.6). To examine the transduction efficiency of manufactured CAR-T products, we performed flow cytometry using a peptide that binds the CD19 CAR (gating scheme depicted in Figure 1B). The median transduction efficiency of CD3^+^ cells was 46.88% (range 29.02–61.09%). Median transduction of CD4 T cells was 57.80% (range 39.40–75.35%) compared to 46.79% (range 24.29–90.38%) for CD8 T cells. The increased transduction rate of CD4 T cells, combined with their greater expansion, led to an overall increase in CAR^+^ CD4 T cells as compared to CAR^+^ CD8 T cells (Figure 1C). All autologous samples were expanded and transduced at sufficient numbers for dosing in the clinical trial (Table 2). The multiplicity of infection of samples was approximately 20, leading to an average estimated vector copy number of 1.89 ± 0.71 per CAR-T cell.

**TABLE 2:** Culture yield, fold expansion, and transduction efficiency.

	**12-day culture**			**8-day culture**		
**Parameter**	**Median culture yield (×10^9^) (*n* = 10)**	**Median fold expansion (*n* = 10)**	**Median CAR^+^ (% of CD45^+^) (*n* = 10)**	**Median culture yield (×10^9^) (*n* = 7)**	**Median fold expansion (*n* = 6)**	**Median CAR^+^ (% of CD45^+^) (*n* = 7)**
CD4	2.22 (0.72–6.5)	51.5 (39.2–212.7)	31.18 (9.0–48.5)	1.0 (0.20–1.4)	24.0 (10.0–48.9)	35.5 (14.8–44.6)
CD8	1.27 (0.56–3.26)	36.0 (8.4–58.7)	13.46 (4.95–28.76)	0.59 (0.05–0.94)	10.9 (0.70–20.5)	14.4 (4.8–35.6)

Following the manufacturer’s protocol, we harvested cells from the Prodigy^®^ on day 12. However, to determine if the time to patient infusion could be shortened, we measured transduction efficiency and cell numbers at earlier time points. On days -1, 3, 6, 9, and 12, we assessed CD3, CD4, CD8, and the CD19 CAR expression by flow cytometry. As represented in Figure 1D, transduction efficiency reached a plateau on day 6. Accordingly, the transduction rate of CD3^+^ cells after 8-day culture was comparable to that at day 12 at a median 44.3% (range 27.6–65.8%). Furthermore, cell counts at day 8 were found to be sufficient to meet the required cell numbers for infusion into patients in our clinical trial exceeding a dose of 1 × 10^6^ transduced CD19 CAR-T cells/kg (Figure 1C and Table 2). We therefore reduced the duration of CAR-T production, and shortened the time from apheresis to treatment.

Product purity is a major concern for the safety and outcome of patients, particularly malignant B cell contamination since a single transduced B cell has been reported to lead to patient relapse (22). The purity of the CD19 CAR-T products was studied by flow cytometry. Samples were stained at harvest for T cells (CD3, CD4, CD8), B cells (CD20), monocytes (CD14), and NK/NKT cells (CD56, CD16). As shown in Figure 1E, there is little to no contamination of B cells (median 0.00%, range 0.00–0.04%), monocytes (median 0.18%, range 0.00–2.15%) and NK cells (median 0.13%, range 0.01–8.57%). Furthermore, the percent viability of CAR-T cells was measured by staining with 7-AAD and products showed a median 93.18% viability (range 86.58–96.25%) (Figure 1F). All products met acceptable standards of purity, viability for patient infusion and cell dose.

### Phenotyping Manufactured CAR-T Product

**FIGURE 2:**
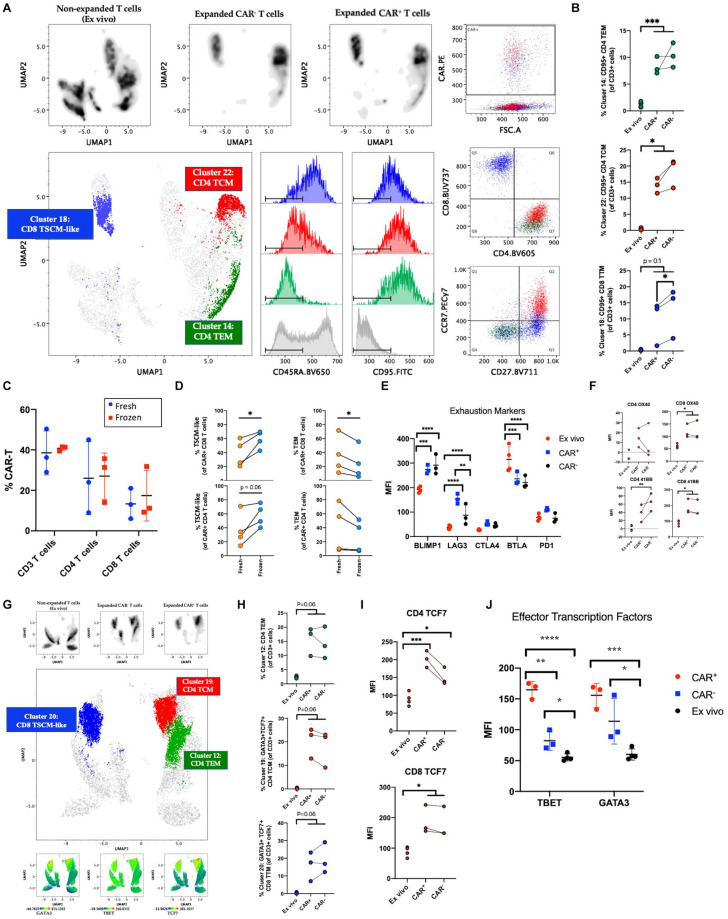
Phenotyping manufactured CAR-T product. **(A)** Clustering analysis of CAR-T cell products (*n* = 3) and freshly isolated T cells (*ex vivo*; *n* = 4) using the markers 41BB, CD8, CCR7, CD27, CD95, CD45RO, CD4, and CD127. Cells were pre-gated on live CD3^+^ singlets prior to analysis. Expanded CAR^+^ and CAR^–^ T cell populations separate by expression pattern from pre-culture T cells. CAR^+^ cells predominantly express markers of stem cell memory (TSCM) among CD8 T cells and central memory (TCM) or effector memory (TEM) among CD4 T cells. **(B)** Percentage of CAR^+^, CAR^–^, and freshly isolated T cells among clusters from **(A)**. **(C)** Percentage of CAR expressing CD3, CD4, and CD8 T cells at harvest (fresh; *n* = 3) and after free-thaw (frozen; *n* = 3). **(D)** Changes in T cell memory subsets after freeze-thaw (*n* = 4). **(E)** Expression of inhibitory checkpoint molecules on *ex vivo* (*n* = 4) or expanded CAR^+^ and CAR^–^ T cells (*n* = 3). **(F)** Expression of OX40 and 41BB prior to culture (*n* = 4) and in post-culture CAR^+^ and CAR^–^ T cells (*n* = 3). **(G)** Clustering analysis of CAR-T cell products (*n* = 3) and freshly isolated T cells (*n* = 4) using the markers CD4, CD95, CD45RO, TBET, GATA3, TCF7, CD8, CCR7, CD27, and CD127. Cells were pre-gated on live CD3^+^ singlets prior to analysis. Expanded CAR^+^ and CAR^–^ T cell populations separate by expression pattern from freshly isolated T cells. Expression of transcription factors GATA3, TBET, and TCF7 overlaps with CAR^+^ cells. **(H)** Percentage of unique phenotypes associated with CAR^+^ T cells of CD3^+^ cells. **(I)** Expression of TCF7 prior to culture (*n* = 4) and in post-culture CAR^+^ and CAR^–^ T cells (*n* = 3). **(J)** Expression of TBET and GATA3 prior to culture (*n* = 4) and in post-culture CAR^+^ and CAR^–^ T cells (*n* = 3). Statistical significance was given as *, **, ***, or **** by *p*-values less than < 0.05, < 0.01, < 0.001, or < 0.0001, respectively.

As previous studies have emphasized, induction of quiescent TSCM and TCM phenotypes in CAR-T cells is desirable for improved self-renewal, effector differentiation, formation of memory, and lowered CRS (4–8, 23). Here, we expected the addition of IL-7/IL-15 in our manufacturing platform to result in a quiescent cellular profile despite extensive activation and 40-fold expansion. To confirm the abundance of quiescent subsets in the CAR-T product, we performed traditional T cell subset gating (Table 3) and observed an enrichment of CD4 and CD8 TSCM cells expressing high levels of CD95 (in addition to CD27 and CCR7) (Supplementary Figures S1A–C). To define unique phenotypes, we performed unbiased clustering using 41BB, CD8, CCR7, CD27, CD95, CD45RO, CD4, and CD127. The CAR-T products showed a distinct profile when compared to freshly isolated T cells while CAR expressing cells showed little variation from CAR^–^ cells within the CAR-T product (Figure 2A). Upon further analysis, we observed three predominant clusters overlap with CAR expression and identified these clusters as CD8 TSCM-like (CD45RA^+^CD27^+^CCR7^–^), CD4 central memory (CD45RA^–^CD27^+^CCR7^+^; TCM) and CD4 effector memory (CD45RA^–^CD27^–^CCR7^–^; TEM) (Figure 2A). Each cluster expressed high levels of CD95 (likely resulting from prolonged exposure to IL-7/IL-15 during manufacturing) and was significantly enhanced among manufactured cells, regardless of transduction status (Figure 2B).

**TABLE 3:** T cell memory subset markers.

**Marker**	**Naive**	**Stem cell memory**	**Central memory**	**Transitional memory**	**Effector memory**	**Terminal effector**
CD45RA	+	+	–	–	–	+
CD45RO	–	–	+	+	+	–
CCR7	+	+	+	–	–	–
CD27	+	+	+	+	–	–
CD127	+	+	+	±	–	–
CD95	–	+	+	+	+	+

As current standard practice is for CAR-T cells to be cryopreserved at harvest and thawed before infusion into patients, we questioned whether freeze-thaw impacts the T cell memory phenotypes distribution observed in the product. The protocol for this clinical trial was to distribute the cells to each patient fresh for infusion, however, two patients received cryopreserved cells due to medical circumstances. While no change was observed in the frequencies of CD4, CD8, or CAR^+^ T cells (Figure 2C and Supplementary Figures S2D–F), our data showed a favorable preservation of TSCM-like cells and loss of TEM (Figure 2D). These data suggest that although freeze-thaw does not alter CAR^+^ cell frequencies or CD4/CD8 proportions, the reduced effector cells post-thawing could contribute to lower incidence of pathologies like CRS because of lower inflammatory response right after product infusion. Successful enrichment of a quiescent profile in the CAR-T cell product after freeze-thaw could promote clinical responses.

Next, to measure immune exhaustion vs. activation status of our product, we studied the levels of several immune checkpoint molecules such as PD1, CTLA4, BTLA, BLIMP1, and LAG3. We observed an increase in BLIMP1 and LAG3 within the product and a decrease of BTLA (Figure 2E and Supplementary Figure S1G). The increase in these markers (also up-regulated upon T cell activation) could be reflective of the highly activated state of the product. In order to further validate the lack of exhaustion, we assessed the relative expression of stemness markers OX40 and 41BB (24–26). As seen in Figure 2F, OX40 and 41BB were upregulated in the product with equivalent expression of 41BB in CAR^+^ and CAR^–^ T cells, excluding the possibility of a vector-driven increase of 41BB. Given that our product showed an enrichment of both stemness and effector surface molecules, we sought to study the heterogeneity in CAR-T clusters using a high-dimensional flow cytometry panel that would show the distribution of transcription factors associated with effector function (TBET, GATA3), and stemness (TCF7) (27–29). To understand the expression of these markers relative to the memory phenotypes we identified earlier, clustering was performed with markers CD4, CD95, CD45RO, TBET, GATA3, TCF7, CD8, CCR7, CD27, and CD127. As seen in Figures 2G,H, expression of TBET, GATA3, and TCF7 colocalize with CAR expression, and GATA3 and TCF7 are specifically enriched among manufactured cells in the CD8 TSCM-like and CD4 TCM clusters. Manual gating on TBET, GATA3, and TCF7 further supports induced expression after culturing conditions and highlights a specific enrichment for CAR^+^ cells among manufactured cells (Figures 2I,J and Supplementary Figure S1H). Altogether, despite higher levels of exhaustion markers, our results indicate the cells have a quiescent profile with the ability to self-renew and is suggestive of a polyfunctional CD4 and CD8 phenotype that is capable of differentiating into effector cells via the expression of stemness and effector transcription factors.

### Manufactured CAR-T Cells Effectively Kill CD19^+^ Cells *in vitro*

**FIGURE 3:**
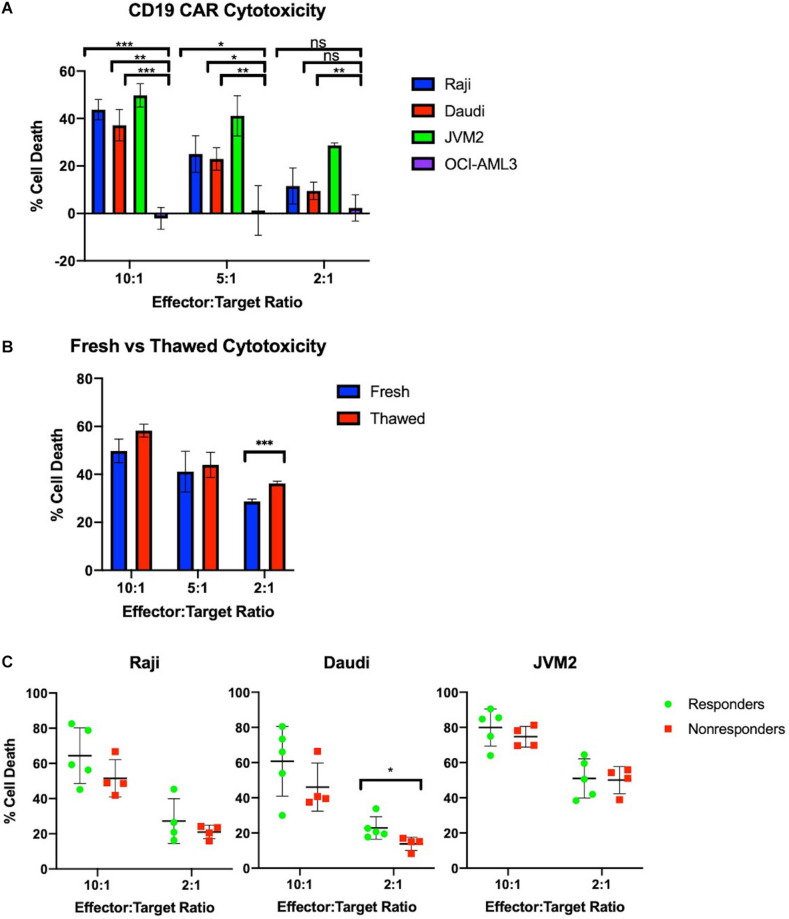
Manufactured CAR-T cells effectively kill CD19^+^ cells *in vitro.* **(A)** CAR-T cells selectively and effectively kill CD19^+^ cells *in vitro* at 10:1 and 5:1 ratios as measured by number of calcein positive target cells compared to target cells alone (*n* = 3). **(B)** Freeze-thaw does not negatively impact JVM2 cell lysis at 10:1, 5:1, and 2:1 ratios (*n* = 3). **(C)** Cytotoxicity of autologous CAR-T cells against CD19^+^ Raji, Daudi, and JVM2 cells stratified by patients with complete response (*n* = 5) or partial response/progressive disease (*n* = 4). Statistical significance was given as *, **, ***, or **** by *p*-values less than < 0.05, < 0.01, < 0.001, or < 0.0001, respectively.

CAR-T cells show a dose-dependent cytotoxicity toward CD19^+^ cancer cells *in vitro*. Effector cell function of the CAR-T samples was tested *in vitro* using a flow cytometry calcein-based cytotoxicity assay (21). Three CD19^+^ B cell lymphoma lines were tested (Raji, Daudi, and JVM2) as well as an acute myeloid leukemia cell line (OCI-AML3) as a CD19^–^ control. Results for cell lysis by three CAR-T samples are shown in Figure 3A. All samples tested showed significantly higher cell lysis of CD19^+^ Raji, Daudi, and JVM2 cells compared to CD19^–^ OCI-AML3 cells at a 10:1 effector to target ratio. Cell lysis decreases in a dose-dependent manner as the effector to target ratio is reduced. Furthermore, as shown in Figure 3B, CAR-T cell cytotoxic activity did not significantly differ when fresh or cryopreserved CAR-T cells were used at all ratios tested.

To evaluate the potency of our products, we tested the CAR-T products of 9 patients prior to infusion against CD19^+^ Raji, Daudi, and JVM2 cell lines at 10:1 and 2:1 ratios; CAR-T products were stratified by complete responders (CR) vs. non-responders (NR) (partial responders or patients with progressive disease). As shown in Figure 3C, CAR-T cells showed high potency across all three cell lines and a minor trend of enhanced cytotoxicity of CR is observed for all three cell lines.

### Manufactured CAR-T Cells Effectively Clear CD19^+^ Tumor Cells *in vivo*

**FIGURE 4:**
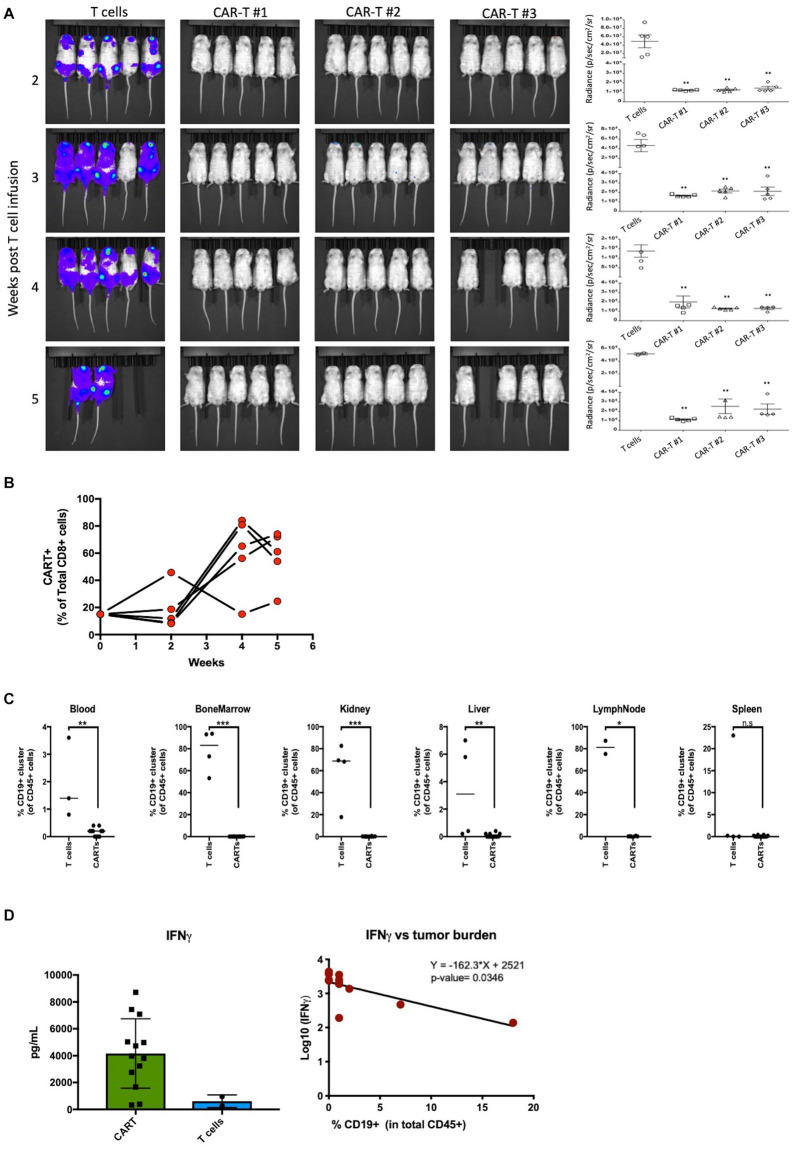
Manufactured CAR-T cells effectively clear CD19^+^ tumor cells *in vivo*. **(A)** Left – Luciferase-expressing Raji cells injected into mice in groups of 5 were treated with CAR-T cells or non-transduced T cell control group and bioluminescence was measured at indicated weeks. Right – Quantification in radiance (p/sec/cm^2^/sr) of tumor burden in mice groups. **(B)** Effector CD8 CAR-T cells expand in mice over 5 weeks in proportion to CAR negative human CD8 T cells. **(C)** Quantification of tumor burden between mice receiving *ex vivo* T cells (*n* = 2–4) or CAR-T cells (*n* = 14) in blood, bone marrow, kidney, liver, lymph nodes, and spleen. **(D)** Mice treated with CAR-T cells have higher levels of effector cytokine IFNγ which negatively correlate with tumor burden. Statistical significance was given as *, **, ***, or **** by *p*-values less than < 0.05, < 0.01, < 0.001, or < 0.0001, respectively.

Effector function of the CAR-T samples was tested *in vivo* in a lymphoma mouse model. NOD-SCID-IL-2 gamma-/- (NSG) mice were injected with luciferase-expressing Raji cells intravenously (*i.v.*) on day 0 and CAR-T cells were injected *i.v.* on day 7. The control mice were injected with comparable non-transduced T cell doses. Mice injected with CAR-T cells showed a significantly (*p* < 0.01) lower tumor burden compared to mice injected with un-transduced T cells as measured by bioluminescent imaging at all weeks tested (Figure 4A). The mice tolerated the CAR-T cells without any apparent toxicities or weight loss (Supplementary Figure S2C). At weeks 2 and 4 post-infusion, mice were bled for quantification of CD8 CAR-T cell expansion by flow cytometric analysis. Our results demonstrate robust expansion of circulating CD8 CAR-T cells by week 4 (Figure 4B). At weeks 5 and 6, mice were sacrificed and organs (spleen, liver, bone marrow, kidney, lymph nodes, and blood) were harvested and homogenized for flow cytometric analysis. As shown in Figure 4C, a Raji tumor cell (CD19^+^CD45^+^) cluster, demarked in an oval, was driven by similar expression of CD19. Quantification of Raji tumor cells demonstrates extensive tumor clearance in all CAR-T treated mouse blood and tissues, whereas tumor burden is present in all organs but the spleen of mice receiving non-transduced T cells (Figure 4D). To assess what cytokines were prevalent in the blood of mice receiving CAR-T cell treatment, we utilized the U-PLEX assay (Supplementary Figure S2A). As expected, we observed increased levels of the CD8 effector cytokine IFNγ in mice receiving CAR-T cells, which significantly correlated with decreased tumor burden (*p*-value = 0.036) (Figure 4D). Complementing the *in vitro* culture results (see Figure 2), our data also show that an increase in IFNγ production was associated with higher levels of TNFα, likely resulting from a polyfunctional CD8 CAR-T cell response (Supplementary Figure S2A). This increase went hand-in-hand with higher levels of innate immune cascades (i.e., higher IL-6 and IL-1b) and of IL-17A and IL-4 (likely a result of CD4 T cell differentiation). A likely counter to this inflammatory response is seen by the induction of TGF-β2 and a decrease in gamma-chain cytokines like IL-2 and IL-7 (Supplementary Figure S2A). Interestingly, of all cytokines measured (Supplementary Figure S2A), TNFα and TGF-β2 also correlated significantly with mouse tumor burden (Supplementary Figure S2B).

### Detection of Manufactured CAR-T Cells in NHL Patients

**FIGURE 5:**
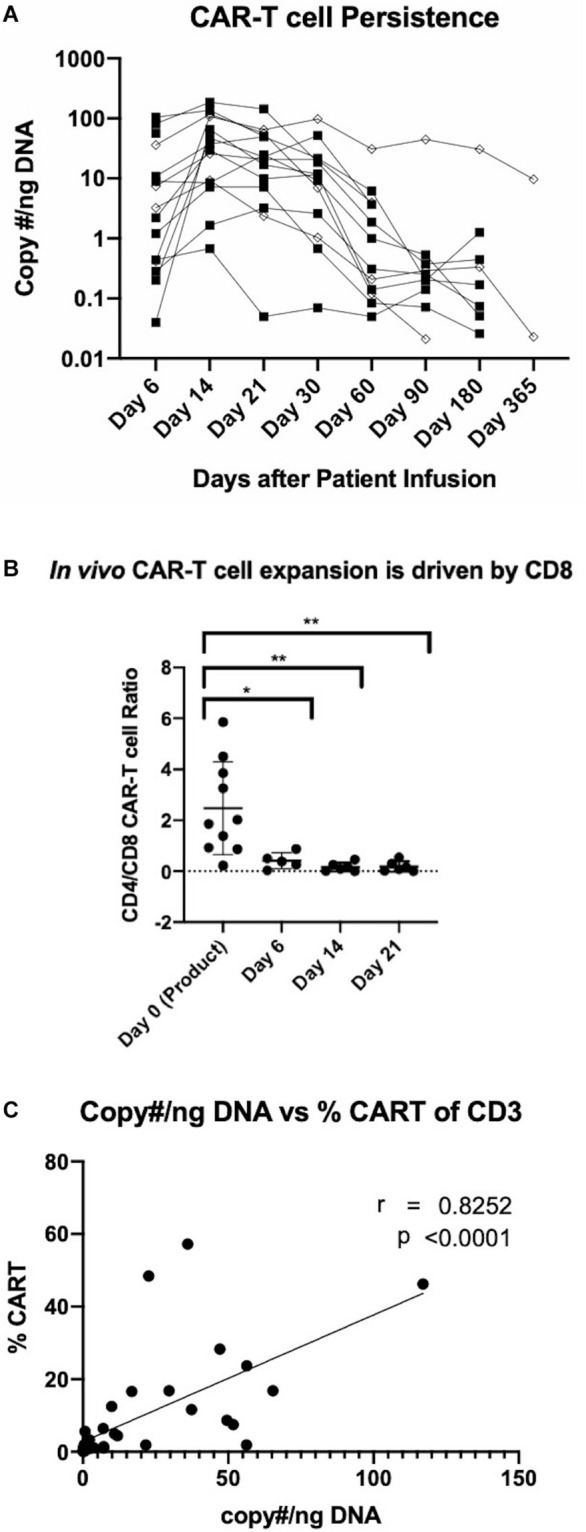
Detection of manufactured CAR-T cells in NHL patients. **(A)** Detection of CAR in patient circulation by PCR for rev-responsive element up to 1 year post-treatment. Diamond and square shapes represent 0.5 × 10^6^ (*n* = 4) and 1.0 × 10^6^ (*n* = 11) CAR-T cells/kg doses, respectively. **(B)** CD4/CD8 CAR-T cell ratios in patients at day 0 (pre-infusion) (*n* = 15), day 6 (*n* = 5), day 14 (*n* = 6) and day 21 (*n* = 6) after treatment. **(C)** Correlation between flow cytometric and RT-qPCR detection of CAR-T cells in patient circulation (*n* = 36). Statistical significance was given as *, **, ***, or **** by *p*-values less than < 0.05, < 0.01, < 0.001, or < 0.0001, respectively.

An evaluation of the safety of anti-CD19 CAR-T cells manufactured using the CliniMACS Prodigy platform is currently underway in a phase I/II clinical trial enrolling patients with relapsed/refractory NHL. To date, patients have been treated with two dose levels at 0.5 × 10^6^ and 1 × 10^6^ CAR-T cells per kg. While a full assessment of patient responses is currently in progress, here we present the persistence of the manufactured CAR-T cells. Peripheral blood samples were obtained at 6, 14, 21, 30, 60, 90, 180, and 365 days after CAR-T cell infusion. Analyses were conducted by qPCR and flow cytometry to quantify CAR-T cell persistence. qPCR analysis was performed by probing for the pro-viral rev-responsive element and is represented by copy number per nanogram of DNA. Flow cytometric analysis of CAR-T cell persistence was performed by staining cells with a FITC-conjugated CD19 peptide. As shown in Figure 5A, patients generally experienced a peak in circulating CAR-T cell levels at day 14 through day 21, with gradual subsequent decreases. Peak expansion or persistence was not affected by cell dose. Flow cytometric quantification of CD8 and CD4 CAR-T cell subsets revealed that despite the predominance of CD4 CAR-T cells in the product, *in vivo* expansion is driven by CD8 CAR-T cells (Figure 5B). CD19 CAR-T cells were detectable by both methods up to 1 year after infusion, and CAR T cells were detected in all patients at all studied time points. Additionally, we demonstrate for the first time the utility of a FITC conjugated CD19 peptide to monitor CD19 CAR-T cells in patient samples and its strong agreement with existing qPCR data (Figure 5C).

## Discussion

Our findings demonstrate that it is possible to robustly manufacture CAR-T cells within a hospital setting using a GMP-compliant closed system. Furthermore, we were able to reduce the turn-around cell manufacturing time to 8 days, lowering the cost and expediting patient treatment. Such fast turn-around time is one of the main advantages of local manufacturing over centralized CAR-T manufacturing strategies. Interestingly, the conditions studied here appear to increase CAR-T cell potency by preventing differentiation and promoting a quiescent/memory profile (14). While our platform allowed patient treatment with fresh CAR-T products, the hypothesis that non-cryopreserved cells might provide better outcomes cannot be answered as we lack a comparison cohort in this study.

In addition to demonstrating the reproducibility of the closed cell manufacturing system, our study emphasizes the need for the standardization of several clinical grade reagents to ensure robust production of CAR-T cells. Unlike previous studies that have reported selective expansion of CD8 CAR-T cells (12, 13), we observed an increase in the frequency of CD4 T cells. This observed difference may be attributable to supplementation with IL-7/IL-15 rather than IL-2, although IL-7/IL-15 stimulation in a similar study led to no noticeable effect on T cell populations [Dominik (30)]. Notably, this affected the final product, with a predominance of CD4 CAR-T. Nonetheless, expansion of CD8 CAR-T cells dominated *in vivo*. In agreement with our findings, previous studies have reported enrichment of a TSCM-like subset from naive T cells upon IL-7/IL-15 supplementation, despite the use of differing gating criteria [(5); Dominik (30)]. In support of this result, we identified increased expression of TCF7, OX40, and 41BB on CAR^+^ cells, indicative of the potential of CAR-T cells for self-renewal and effector differentiation. These CAR expressing cells that maintained a TCM/TSCM (CD27+) phenotype had higher levels of CD95, suggestive of an activated state. The activated state of these cells was further confirmed by an increase in the canonical Th1 and Th2 transcription factors TBET and GATA3, and an induction of activation-associated upregulation of LAG3, CTLA4, and PD1. The cell subset described here is compatible with both effector and stemness properties; together these qualities could be crucial to safe and long-lasting responses.

Another technical challenge addressed by our study is the development of a flow cytometric assay to monitor the persistence of circulating CAR-T cells in patients. While there are available reagents such as a monoclonal antibody detecting the scFv region of the FMC63 CD19 CAR (31), protein L (32), or fluorescent-tags, these assays are not commercially available, lack specificity, or are technically challenging in the clinical setting. Here, we employed a commercially available FITC-conjugated CD19 peptide with great success as demonstrated by a strong correlation between detection of circulating CAR-T cells in patient blood samples by viral qPCR and flow cytometry.

Since a major hurdle in the CAR-T cell therapy is the ability to predict the effectiveness of CAR-T products, we performed a potency assay (cytotoxicity assay) and correlated with clinical responses. Though a larger number of patients will be needed to appreciate its value, a subtle pattern was observed whereby the CAR-T products of patients achieving CR had greater cytotoxicity. This indicates the presence of intrinsic differences in CAR-T products which may affect response to CAR-T cell therapy. Recent studies have suggested various factors that may influence CAR-T cell response, such as age or the exhaustion phenotype of the starting sample (33, 34). Thus, our results warrant further investigation of the differences between CAR-T of responding and non-responding patients, particularly through functional assays or at the single cell level to understand product heterogeneity.

## Conclusion

In conclusion, we report an efficient means to reproducibility manufacture functional autologous CD19 CAR-T cells for clinical application. We demonstrate co-stimulation of patient-derived T cells with IL-7 and IL-15 promotes a TSCM-like phenotype that is efficacious in the lysis of CD19 expressing cells *in vitro*, *in vivo*, and in the clinic. CAR-T cells persisted *in vivo* up to 6 weeks in our mouse tumor model and were detectable in circulation of patients up to 1 year after infusion. Lastly, we provide manufacturing benchmark numbers for inter-institutional comparisons of this approach in the future.

## Data Availability Statement

The raw data supporting the conclusions of this article will be made available upon request by the authors, without undue reservation.

## Ethics Statement

The studies involving human participants were reviewed and approved by the University Hospitals Seidman Cancer Center Institutional Review Board. The patients/participants provided their written informed consent to participate in this study. The animal study was reviewed and approved by the Case Western Reserve University Institutional Animal Care and Use Committee.

## Author Contributions

ZJ and AR wrote the manuscript. ZJ, AR, AS, and FL analyzed the data. ZJ, AR, AS, FL, and SM designed and performed the experiments. DS and RO invented the CAR construct utilized in this study. YX performed the experiments. DS and YX developed the CAR staining protocol and contributed to the development of CliniMACS Prodigy^®^ procedures. AR and RL performed the animal study. SK-B and JR developed the Prodigy manufacture for this CAR-T product and the procedure for 8 day culture. SK-B, JR, and KZ developed the *in vivo* CAR-T staining. KZ and JS performed CAR-T detection studies. DW, ML, and R-PS designed and supervised the study. DW and ML funded the experiments. All authors contributed to the article and approved the submitted version.

## Conflict of Interest

YX, WK, AW, MK, DS, and BD are employed at Lentigen, a Miltenyi Biotec Company.

The remaining authors declare that the research was conducted in the absence of any commercial or financial relationships that could be construed as a potential conflict of interest.

## Footnotes

1 https://rstudio.com

2 https://microsoft.com

## References

[1] Mohty M Gautier J Malard F Aljurf M Bazarbachi A Chabannon C CD19 chimeric antigen receptor-T cells in B-cell leukemia and lymphoma: current status and perspectives. *Leukemia.* (2019) 33:2767–78. 10.1038/s41375-019-0615-5 31690821

[2] Hao L Li T Chang LJ Chen X. Adoptive immunotherapy for B-cell malignancies using CD19- targeted chimeric antigen receptor T-cells: a systematic review of efficacy and safety. *Curr Med Chem.* (2019) 26:3068–79. 10.2174/0929867324666170801101842 28762313

[3] Khan JF Khan AS Brentjens RJ. Application of CAR T cells for the treatment of solid tumors. *Prog Mol Biol Transl Sci.* (2019) 164:293–327. 10.1016/bs.pmbts.2019.07.004 31383408

[4] Gattinoni L Lugli E Ji Y Pos Z Paulos CM Quigley MF A human memory T cell subset with stem cell-like properties. *Nat Med.* (2011) 17:1290–7. 10.1038/nm.2446 21926977 PMC3192229

[5] Cieri N Camisa B Cocchiarella F Forcato M Oliveira G Provasi E IL-7 and IL-15 instruct the generation of human memory stem T cells from naive precursors. *Blood.* (2013) 121:573–84. 10.1182/blood-2012-05-431718 23160470

[6] Lugli E Dominguez MH Gattinoni L Chattopadhyay PK Bolton DL Song K Superior T memory stem cell persistence supports long-lived T cell memory. *J Clin Invest.* (2013) 123:594–9. 10.1172/JCI66327 23281401 PMC3561805

[7] Biasco L Scala S Basso Ricci L Dionisio F Baricordi C Calabria A In vivo tracking of T cells in humans unveils decade-long survival and activity of genetically modified T memory stem cells. *Sci Transl Med.* (2015) 7:273ra213. 10.1126/scitranslmed.3010314 25653219

[8] Wang X Popplewell LL Wagner JR Naranjo A Blanchard MS Mott MR Phase 1 studies of central memory-derived CD19 CAR T-cell therapy following autologous HSCT in patients with B-cell NHL. *Blood.* (2016) 127:2980–90. 10.1182/blood-2015-12-686725 27118452 PMC4911862

[9] Wang X Riviere I. Clinical manufacturing of CAR T cells: foundation of a promising therapy. *Mol Ther Oncolytics.* (2016) 3:16015. 10.1038/mto.2016.15 27347557 PMC4909095

[10] Mock U Nickolay L Philip B Cheung GW Zhan H Johnston ICD Automated manufacturing of chimeric antigen receptor T cells for adoptive immunotherapy using CliniMACS prodigy. *Cytotherapy.* (2016) 18:1002–11. 10.1016/j.jcyt.2016.05.009 27378344

[11] Priesner C Aleksandrova K Esser R Mockel-Tenbrinck N Leise J Drechsel K Automated enrichment, transduction, and expansion of clinical-scale CD62L(+) T cells for manufacturing of gene therapy medicinal products. *Hum Gene Ther.* (2016) 27:860–9. 10.1089/hum.2016.091 27562135 PMC5035932

[12] Zhang W Jordan KR Schulte B Purev E. Characterization of clinical grade CD19 chimeric antigen receptor T cells produced using automated CliniMACS Prodigy system. *Drug Des Devel Ther.* (2018) 12:3343–56. 10.2147/DDDT.S175113 30323566 PMC6181073

[13] Zhu F Shah N Xu H Schneider D Orentas R Dropulic B Closed-system manufacturing of CD19 and dual-targeted CD20/19 chimeric antigen receptor T cells using the CliniMACS Prodigy device at an academic medical center. *Cytotherapy.* (2018) 20:394–406. 10.1016/j.jcyt.2017.09.005 29287970

[14] Ghassemi S Nunez-Cruz S O’Connor RS Fraietta JA Patel PR Scholler J Reducing Ex Vivo culture improves the antileukemic activity of chimeric antigen receptor (CAR) T cells. *Cancer Immunol Res.* (2018) 6:1100–9. 10.1158/2326-6066.CIR-17-0405 30030295 PMC8274631

[15] Xia A Zhang Y Xu J Yin T Lu XJ. T Cell dysfunction in cancer immunity and immunotherapy. *Front Immunol.* (2019) 10:1719. 10.3389/fimmu.2019.01719 31379886 PMC6659036

[16] Sommermeyer D Hudecek M Kosasih PL Gogishvili T Maloney DG Turtle CJ Chimeric antigen receptor-modified T cells derived from defined CD8+ and CD4+ subsets confer superior antitumor reactivity in vivo. *Leukemia.* (2016) 30:492–500. 10.1038/leu.2015.247 26369987 PMC4746098

[17] Fraietta JA Lacey SF Orlando EJ Pruteanu-Malinici I Gohil M Lundh S Determinants of response and resistance to CD19 chimeric antigen receptor (CAR) T cell therapy of chronic lymphocytic leukemia. *Nat Med.* (2018) 24:563–71. 10.1038/s41591-018-0010-1 29713085 PMC6117613

[18] Schneider D Xiong Y Wu D Nlle V Schmitz S Haso W A tandem CD19/CD20 CAR lentiviral vector drives on-target and off-target antigen modulation in leukemia cell lines. *J Immunother Cancer.* (2017) 5:42. 10.1186/s40425-017-0246-1 28515942 PMC5433150

[19] Levine JH Simonds EF Bendall SC Davis KL Amir ED Tadmor MD Data-driven phenotypic dissection of AML reveals progenitor-like cells that correlate with prognosis. *Cell.* (2015) 162:184–97. 10.1016/j.cell.2015.05.047 26095251 PMC4508757

[20] McInnes L Healy J Saul N Großberger L. UMAP: uniform manifold approximation and projection. *J Open Source Softw.* (2018) 3:861 10.21105/joss.00861

[21] Ojo EO Sharma AA Liu R Moreton S Checkley-Luttge MA Gupta K Membrane bound IL-21 based NK cell feeder cells drive robust expansion and metabolic activation of NK cells. *Sci Rep.* (2019) 9:14916. 10.1038/s41598-019-51287-6 31624330 PMC6797802

[22] Ruella M Xu J Barrett DM Fraietta JA Reich TJ Ambrose DE Induction of resistance to chimeric antigen receptor T cell therapy by transduction of a single leukemic B cell. *Nat Med.* (2018) 24:1499–503. 10.1038/s41591-018-0201-9 30275568 PMC6511988

[23] Zhou J Jin L Wang F Zhang Y Liu B Zhao T. Chimeric antigen receptor T (CAR-T) cells expanded with IL-7/IL-15 mediate superior antitumor effects. *Protein Cell.* (2019) 10:764–9. 10.1007/s13238-019-0643-y 31250350 PMC6776495

[24] Redmond WL Ruby CE Weinberg AD. The role of OX40-mediated co-stimulation in T-cell activation and survival. *Crit Rev Immunol.* (2009) 29:187–201. 10.1615/critrevimmunol.v29.i3.10 19538134 PMC3180959

[25] Bartkowiak T Curran MA. 4-1BB agonists: multi-potent potentiators of tumor immunity. *Front Oncol.* (2015) 5:117. 10.3389/fonc.2015.00117 26106583 PMC4459101

[26] Sanchez-Paulete AR Labiano S Rodriguez-Ruiz ME Azpilikueta A Etxeberria I Bolanos E Deciphering CD137 (4-1BB) signaling in T-cell costimulation for translation into successful cancer immunotherapy. *Eur J Immunol.* (2016) 46:513–22. 10.1002/eji.201445388 26773716

[27] Gattinoni L Zhong XS Palmer DC Ji Y Hinrichs CS Yu Z Wnt signaling arrests effector T cell differentiation and generates CD8+ memory stem cells. *Nat Med.* (2009) 15:808–13. 10.1038/nm.1982 19525962 PMC2707501

[28] Jeannet G Boudousquie C Gardiol N Kang J Huelsken J Held W. Essential role of the Wnt pathway effector Tcf-1 for the establishment of functional CD8 T cell memory. *Proc Natl Acad Sci USA.* (2010) 107:9777–82. 10.1073/pnas.0914127107 20457902 PMC2906901

[29] Zhou X Yu S Zhao DM Harty JT Badovinac VP Xue HH. Differentiation and persistence of memory CD8(+) T cells depend on T cell factor 1. *Immunity.* (2010) 33:229–40. 10.1016/j.immuni.2010.08.002 20727791 PMC2928475

[30] Lock D Mockel-Tenbrinck N Drechsel K Barth C Mauer D Schaser T Automated manufacturing of potent CD20-directed chimeric antigen receptor T cells for clinical use. *Hum Gene Ther.* (2017) 28:914–25. 10.1089/hum.2017.111 28847167

[31] Jena B Maiti S Huls H Singh H Lee DA Champlin RE Chimeric antigen receptor (CAR)-specific monoclonal antibody to detect CD19-specific T cells in clinical trials. *PLoS One.* (2013) 8:e57838. 10.1371/journal.pone.0057838 23469246 PMC3585808

[32] Zheng Z Chinnasamy N Morgan RA. Protein L: a novel reagent for the detection of chimeric antigen receptor (CAR) expression by flow cytometry. *J Transl Med.* (2012) 10:29. 10.1186/1479-5876-10-29 22330761 PMC3299624

[33] Park JH Riviere I Gonen M Wang X Senechal B Curran KJ Long-term follow-up of CD19 CAR therapy in acute lymphoblastic leukemia. *N Engl J Med.* (2018) 378:449–59. 10.1056/NEJMoa1709919 29385376 PMC6637939

[34] Finney OC Brakke HM Rawlings-Rhea S Hicks R Doolittle D Lopez M CD19 CAR T cell product and disease attributes predict leukemia remission durability. *J Clin Invest.* (2019) 129:2123–32. 10.1172/JCI125423 30860496 PMC6486329

