# Supplementary figures and images for: Automated Manufacture of Autologous CD19 CAR-T Cells for Treatment of Non-hodgkin Lymphoma
Source: Front Immunol. 2020 Aug 7;11:1941. doi: 10.3389/fimmu.2020.01941 (PMC7427107; doi:10.3389/fimmu.2020.01941)

## Slide 1
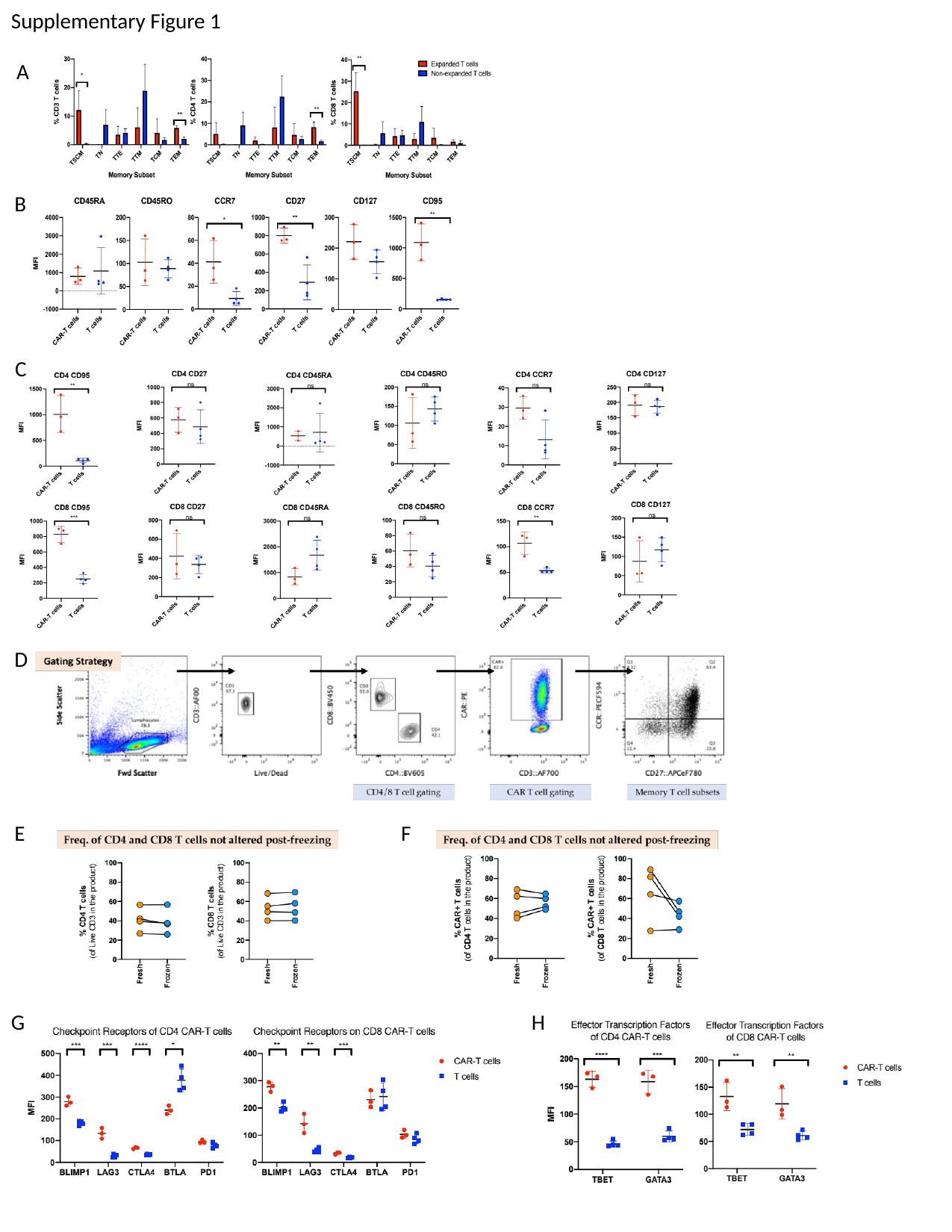

Supplementary Figure 1
A
B
C
D
F
E
G
H

## Slide 2
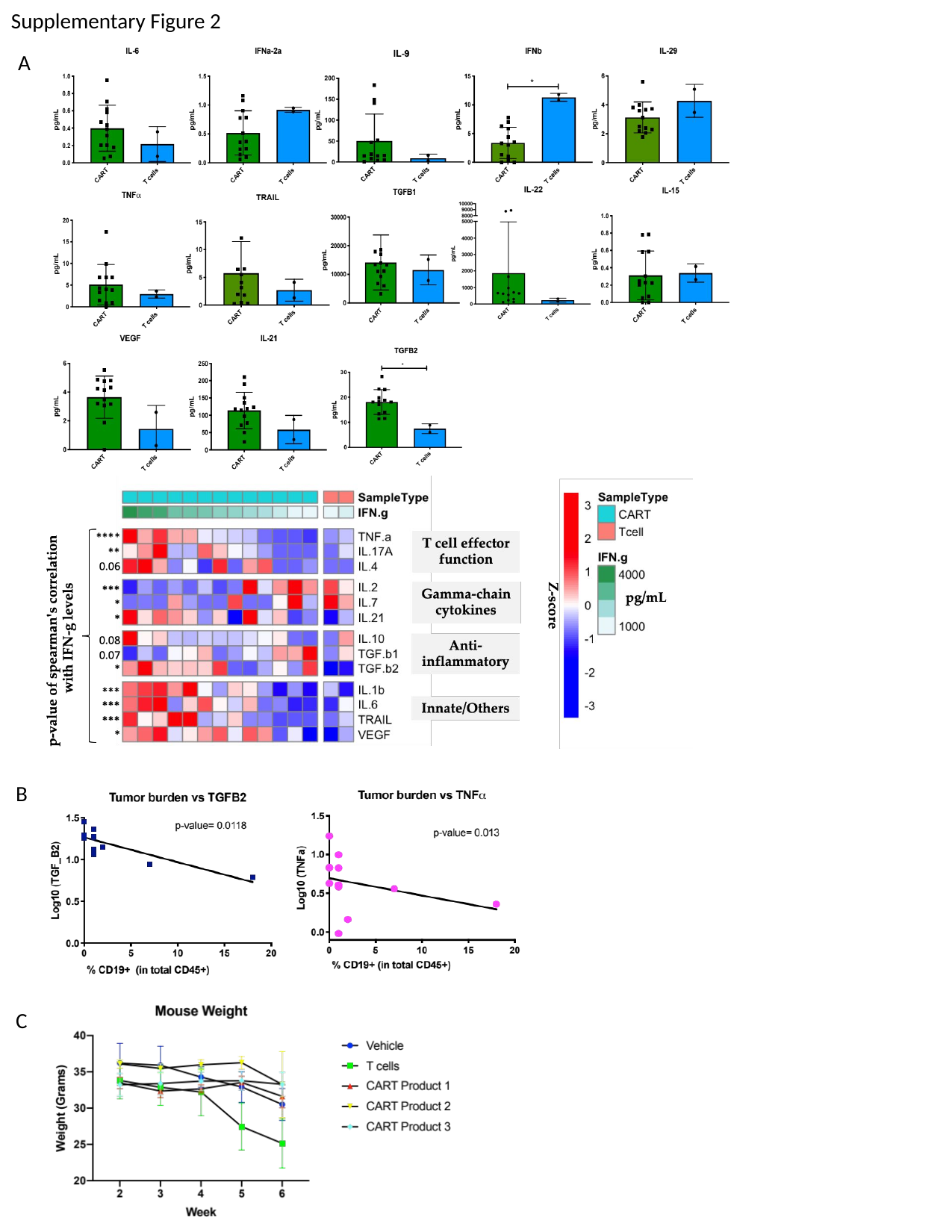

Supplementary Figure 2
A
B
C

Supplement: Supplementary file 1 [file Presentation_1.PPTX]
